# Supplementary material for: Molecular Characterization of the Response to Conventional Chemotherapeutics in Pro-B-ALL Cell Lines in Terms of Tumor Relapse
Source: Genes (Basel). 2022 Jul 14;13(7):1240. doi: 10.3390/genes13071240 (PMC9316692; doi:10.3390/genes13071240)
Supplement: Supplementary file 1 [file genes-13-01240-s001.zip › Supplement file S1.pdf]

Supplementary File S1

Molecular Characterization of the Response to Conventional Chemotherapeutics in Pro-B-ALL Cell Lines in Terms of Tumor Relapse

Yvonne Saara Gladbach [1,2,3,+], Lisa-Madeleine Sklarz [4,+], Catrin Roolf [4], Julia Beck [5], Ekkehard Schütz [5], Georg Fuellen [1], Christian Junghanss [4], Hugo Murua Escobar [4,6] and Mohamed Hamed [1,*]

Supplementary Tables

Supplementary Table S1. Gene Ontology terms related to stress release and metabolism as reported in DAVID. This list was used to filter out the DEG sets.

Gene Ontology IDs and terms
GO:0000302~response to reactive oxygen species
GO:0001101~response to acid chemical
GO:0001501~skeletal system development
GO:0001816~cytokine production
GO:0001817~regulation of cytokine production
GO:0001819~positive regulation of cytokine production
GO:0005975~carbohydrate metabolic process
GO:0006000~fructose metabolic process
GO:0006022~aminoglycan metabolic process
GO:0006029~proteoglycan metabolic process
GO:0006082~organic acid metabolic process
GO:0006090~pyruvate metabolic process
GO:0006140~regulation of nucleotide metabolic process
GO:0006164~purine nucleotide biosynthetic process
GO:0006171~cAMP biosynthetic process
GO:0006259~DNA metabolic process
GO:0006575~cellular modified amino acid metabolic process
GO:0006730~one-carbon metabolic process
GO:0006733~oxidoreduction coenzyme metabolic process
GO:0006757~ATP generation from ADP
GO:0006760~folic acid-containing compound metabolic process
GO:0006793~phosphorus metabolic process
GO:0006796~phosphate-containing compound metabolic process

**GO:0006809~nitric oxide biosynthetic process**
**GO:0006950~response to stress**
**GO:0006974~cellular response to DNA damage stimulus**
**GO:0006979~response to oxidative stress**
**GO:0008652~cellular amino acid biosynthetic process**
**GO:0009124~nucleoside monophosphate biosynthetic process**
**GO:0009127~purine nucleoside monophosphate biosynthetic process**
**GO:0009132~nucleoside diphosphate metabolic process**
**GO:0009135~purine nucleoside diphosphate metabolic process**
**GO:0009142~nucleoside triphosphate biosynthetic process**
**GO:0009145~purine nucleoside triphosphate biosynthetic process**
**GO:0009152~purine ribonucleotide biosynthetic process**
**GO:0009156~ribonucleoside monophosphate biosynthetic process**
**GO:0009163~nucleoside biosynthetic process**
**GO:0009165~nucleotide biosynthetic process**
**GO:0009168~purine ribonucleoside monophosphate biosynthetic process**
**GO:0009179~purine ribonucleoside diphosphate metabolic process**
**GO:0009185~ribonucleoside diphosphate metabolic process**
**GO:0009190~cyclic nucleotide biosynthetic process**
**GO:0009260~ribonucleotide biosynthetic process**
**GO:0009605~response to external stimulus**
**GO:0009607~response to biotic stimulus**
**GO:0009628~response to abiotic stimulus**
**GO:0009719~response to endogenous stimulus**
**GO:0009743~response to carbohydrate**
**GO:0009891~positive regulation of biosynthetic process**
**GO:0009892~negative regulation of metabolic process**
**GO:0009893~positive regulation of metabolic process**
**GO:0009991~response to extracellular stimulus**
**GO:0010033~response to organic substance**
**GO:0010035~response to inorganic substance**
**GO:0010243~response to organonitrogen compound**
**GO:0010562~positive regulation of phosphorus metabolic process**
**GO:0010563~negative regulation of phosphorus metabolic process**
**GO:0010604~positive regulation of macromolecule metabolic process**
**GO:0010605~negative regulation of macromolecule metabolic process**
**GO:0014070~response to organic cyclic compound**
**GO:0016052~carbohydrate catabolic process**
**GO:0016053~organic acid biosynthetic process**
**GO:0019220~regulation of phosphate metabolic process**
**GO:0019222~regulation of metabolic process**
**GO:0019362~pyridine nucleotide metabolic process**
**GO:0019751~polyol metabolic process**

GO:0019752~carboxylic acid metabolic process
GO:0030203~glycosaminoglycan metabolic process
GO:0030799~regulation of cyclic nucleotide metabolic process
GO:0030801~positive regulation of cyclic nucleotide metabolic process
GO:0030802~regulation of cyclic nucleotide biosynthetic process
GO:0030804~positive regulation of cyclic nucleotide biosynthetic process
GO:0030808~regulation of nucleotide biosynthetic process
GO:0030810~positive regulation of nucleotide biosynthetic process
GO:0030814~regulation of cAMP metabolic process
GO:0030816~positive regulation of cAMP metabolic process
GO:0030817~regulation of cAMP biosynthetic process
GO:0030819~positive regulation of cAMP biosynthetic process
GO:0031098~stress-activated protein kinase signaling cascade
GO:0031323~regulation of cellular metabolic process
GO:0031324~negative regulation of cellular metabolic process
GO:0031325~positive regulation of cellular metabolic process
GO:0031328~positive regulation of cellular biosynthetic process
GO:0032101~regulation of response to external stimulus
GO:0032102~negative regulation of response to external stimulus
GO:0032268~regulation of cellular protein metabolic process
GO:0032269~negative regulation of cellular protein metabolic process
GO:0032270~positive regulation of cellular protein metabolic process
GO:0032787~monocarboxylic acid metabolic process
GO:0032872~regulation of stress-activated MAPK cascade
GO:0032874~positive regulation of stress-activated MAPK cascade
GO:0033554~cellular response to stress
GO:0034097~response to cytokine
GO:0034405~response to fluid shear stress
GO:0034599~cellular response to oxidative stress
GO:0034614~cellular response to reactive oxygen species
GO:0035690~cellular response to drug
GO:0036293~response to decreased oxygen levels
GO:0036294~cellular response to decreased oxygen levels
GO:0042221~response to chemical
GO:0042398~cellular modified amino acid biosynthetic process
GO:0042451~purine nucleoside biosynthetic process
GO:0042455~ribonucleoside biosynthetic process
GO:0042493~response to drug
GO:0042558~pteridine-containing compound metabolic process
GO:0043068~positive regulation of programmed cell death
GO:0043086~negative regulation of catalytic activity
GO:0043207~response to external biotic stimulus
GO:0043436~oxoacid metabolic process

GO:0044236~multicellular organism metabolic process
GO:0044262~cellular carbohydrate metabolic process
GO:0044281~small molecule metabolic process
GO:0044710~single-organism metabolic process
GO:0044711~single-organism biosynthetic process
GO:0044723~single-organism carbohydrate metabolic process
GO:0045935~positive regulation of nucleobase-containing compound metabolic process
GO:0045936~negative regulation of phosphate metabolic process
GO:0045937~positive regulation of phosphate metabolic process
GO:0045978~negative regulation of nucleoside metabolic process
GO:0045981~positive regulation of nucleotide metabolic process
GO:0046031~ADP metabolic process
GO:0046033~AMP metabolic process
GO:0046058~cAMP metabolic process
GO:0046129~purine ribonucleoside biosynthetic process
GO:0046209~nitric oxide metabolic process
GO:0046390~ribose phosphate biosynthetic process
GO:0046496~nicotinamide nucleotide metabolic process
GO:0046653~tetrahydrofolate metabolic process
GO:0046655~folic acid metabolic process
GO:0046939~nucleotide phosphorylation
GO:0048519~negative regulation of biological process
GO:0048583~regulation of response to stimulus
GO:0048584~positive regulation of response to stimulus
GO:0048585~negative regulation of response to stimulus
GO:0050790~regulation of catalytic activity
GO:0050896~response to stimulus
GO:0051052~regulation of DNA metabolic process
GO:0051053~negative regulation of DNA metabolic process
GO:0051054~positive regulation of DNA metabolic process
GO:0051094~positive regulation of developmental process
GO:0051129~negative regulation of cellular component organization
GO:0051173~positive regulation of nitrogen compound metabolic process
GO:0051174~regulation of phosphorus metabolic process
GO:0051246~regulation of protein metabolic process
GO:0051247~positive regulation of protein metabolic process
GO:0051248~negative regulation of protein metabolic process
GO:0051403~stress-activated MAPK cascade
GO:0051716~cellular response to stimulus
GO:0052652~cyclic purine nucleotide metabolic process
GO:0055114~oxidation-reduction process
GO:0060255~regulation of macromolecule metabolic process

GO:0070302~regulation of stress-activated protein kinase signaling cascade
GO:0070304~positive regulation of stress-activated protein kinase signaling cascade
GO:0070482~response to oxygen levels
GO:0070887~cellular response to chemical stimulus
GO:0071216~cellular response to biotic stimulus
GO:0071229~cellular response to acid chemical
GO:0071310~cellular response to organic substance
GO:0071345~cellular response to cytokine stimulus
GO:0071407~cellular response to organic cyclic compound
GO:0071417~cellular response to organonitrogen compound
GO:0071453~cellular response to oxygen levels
GO:0071495~cellular response to endogenous stimulus
GO:0071897~DNA biosynthetic process
GO:0072522~purine-containing compound biosynthetic process
GO:0072524~pyridine-containing compound metabolic process
GO:0072593~reactive oxygen species metabolic process
GO:0080090~regulation of primary metabolic process
GO:0080134~regulation of response to stress
GO:0080135~regulation of cellular response to stress
GO:0090407~organophosphate biosynthetic process
GO:1900371~regulation of purine nucleotide biosynthetic process
GO:1900373~positive regulation of purine nucleotide biosynthetic process
GO:1900542~regulation of purine nucleotide metabolic process
GO:1900544~positive regulation of purine nucleotide metabolic process
GO:1901031~regulation of response to reactive oxygen species
GO:1901135~carbohydrate derivative metabolic process
GO:1901136~carbohydrate derivative catabolic process
GO:1901137~carbohydrate derivative biosynthetic process
GO:1901293~nucleoside phosphate biosynthetic process
GO:1901564~organonitrogen compound metabolic process
GO:1901565~organonitrogen compound catabolic process
GO:1901566~organonitrogen compound biosynthetic process
GO:1901615~organic hydroxy compound metabolic process
GO:1901617~organic hydroxy compound biosynthetic process
GO:1901698~response to nitrogen compound
GO:1901700~response to oxygen-containing compound
GO:1901701~cellular response to oxygen-containing compound
GO:1902882~regulation of response to oxidative stress
GO:1903409~reactive oxygen species biosynthetic process
GO:1903510~mucopolysaccharide metabolic process
GO:1903579~negative regulation of ATP metabolic process
GO:2001057~reactive nitrogen species metabolic process

**GO:2001169~regulation of ATP biosynthetic process**
**GO:2001171~positive regulation of ATP biosynthetic process**

Supplementary Table S2. Number of DEGs before and after filtration for drug/stress release and metabolism. The DEGs are based on a p-value of $p < 0.05$, DEGs are calculated for the two pro-B-ALL cell lines after exposition to AraC and Dexa.

**Agent**	**RS4;11**	**SEM**
**before filtration**		
**AraC**	776	927
**Dexa**	3357	1025
**after filtration**		
**AraC**	353	366
**Dexa**	2183	379

Supplementary Table S3. miRNA enrichment for the AraC drug release in RS4;11. Based on a p-value of $p < 0.05$ the miRNA enrichment was used to identify disease and functional annotations.

**Disease/ Ontology**	**adjusted p-value**
**Lymphoma, Primary Effusion**	9.00E-13
**Leukemia, Myeloid, Acute**	0.0000133
**Leukemia, Lymphoblastic, Acute, Childhood**	0.000151
**Leukemia, Myeloid, Chronic**	0.000164
**Leukemia, Myeloid**	0.000839
**Lymphoma, Hodgkin**	0.00187
**Acute Megakaryoblastic Leukemia**	0.00187
**Leukemia, Promyelocytic, Acute**	0.00562
**Leukemia, Biphenotypic, Acute**	0.00893
**Leukemia-Lymphoma, Precursor B-Cell Lymphoblastic**	0.0204
**Leukemia, B-Cell**	0.0477
**Tumor Suppressor miRNAs**	5.21E-11
**Cell Differentiation**	1.16E-10
**Cell Death**	7.30E-10
**Folliculogenesis**	1.09E-09
**Innate Immunity**	2.16E-08
**Glucose Metabolism**	1.55E-07

**Adipocyte Differentiation**	2.76E-07
**Anti-Cell Proliferation**	4.19E-07
**Hematopoiesis**	4.95E-07
**Cell Division**	0.00000173
**Cell Proliferation**	0.0000129
**T-Cell Differentiation**	0.0000282
**Epithelial-to-Mesenchymal Transition**	0.000126
**Hepatic Stellate Cell Differentiation**	0.000147
**Neural Stem Cell Differentiation**	0.000289
**Osteoblast Differentiation**	0.000292
**Aging**	0.000721
**Cell Cycle**	0.000749
**Mesenchymal Stem Cell Proliferation**	0.000781
**Inflammation**	0.00127
**Cardiac Regeneration**	0.00187
**Hormone-mediated Signaling Pathway**	0.00269
**Megakaryocyte Differentiation**	0.00605
**Immune Response**	0.00675
**Apoptosis**	0.0145
**Plasma Cell Differentiation**	0.0161
**Wound Healing**	0.019
**Lipid Metabolism**	0.0251
**Bone Regeneration**	0.0386
**Carbohydrate Metabolism**	0.2088

Supplementary Table S4. miRNA enrichment for the Dexa drug release in RS4;11. Based on a p-value of $p < 0.05$ the miRNA enrichment was used to identify disease and functional annotation for the Dexamethasone drug release in RS4;11.

**Disease/ Ontology**	**adjusted p-value**
**Lymphoma, Primary Effusion**	1.26E-13
**Leukemia, Myeloid, Acute**	3.81E-09
**Leukemia, Myeloid, Chronic**	5.26E-08
**Lymphoma, Hodgkin**	1.57E-07
**Leukemia, Lymphocytic, Chronic**	3.00E-06
**Leukemia, Lymphoblastic, Acute**	5.73E-06
**Leukemia**	3.09E-05
**Leukemia, Lymphoblastic, Acute, T-Cell**	4.18E-04

**Leukemia, Lymphocytic, Chronic, B-Cell**	6.00E-04
**Lymphoma, Mantle-Cell**	6.24E-04
**Lymphoma, Large B-Cell**	1.07E-03
**Lymphoma, B-Cell**	6.73E-03
**Leukemia, Lymphoblastic**	0.0294
**Cell Differentiation**	2.97E-21
**Tumor Suppressor miRNAs**	1.16E-16
**Cell Division**	1.58E-15
**Cell Death**	6.50E-15
**Cell Cycle**	2.47E-14
**Aging**	3.04E-14
**Epithelial-to-Mesenchymal Transition**	6.07E-12
**Cell Proliferation**	4.24E-11
**T-Cell Differentiation**	1.77E-10
**Anti-Cell Proliferation**	1.95E-10
**Apoptosis**	5.10E-10
**Hormone-mediated Signaling Pathway**	6.08E-10
**Neuron Differentiation**	1.78E-09
**Glucose Metabolism**	4.08E-09
**Adipocyte Differentiation**	1.89E-08
**Innate Immunity**	3.30E-07
**Folliculogenesis**	3.63E-07
**Hematopoiesis**	7.63E-07
**Immune Response**	2.66E-06
**Inflammation**	4.50E-06
**Osteogenesis**	9.45E-06
**Regulation of NF-κB Pathway**	3.34E-05
**Bile Duct Proliferation**	3.35E-05
**Neutrophil Differentiation**	3.35E-05
**Cardiac Regeneration**	4.47E-05
**Regulation of Stem Cell**	1.35E-04
**Hepatic Stellate Cell Differentiation**	3.19E-04
**Muscle Development**	4.20E-04
**Circadian Rhythm**	5.31E-04
**Neural Stem Cell Differentiation**	6.24E-04
**Regulation of WNT Signaling Pathway**	6.24E-04
**Osteoclastogenesis**	6.24E-04
**Peritoneal Cavity Homeostasis**	6.62E-04
**Brain Development**	8.27E-04
**Angiogenesis**	8.71E-04
**Osteoblast Differentiation**	9.96E-04
**Neuron Apoptosis**	1.07E-03

**DNA Damage Response**	1.40E-03
**Transdifferentiation**	1.67E-03
**Embryonic Development**	1.78E-03
**Smooth Muscle Cell Proliferation**	2.24E-03
**Cardiomyocyte Differentiation**	3.13E-03
**Granulopoiesis**	3.41E-03
**Cell Motility**	4.07E-03
**Skeletal Muscle Cell Differentiation**	4.86E-03
**Cardiomyocyte Proliferation**	6.12E-03
**Osteoclast Differentiation**	9.44E-03
**Natural Killer Cell Activation**	9.99E-03
**Chondrogenic Differentiation**	0.0147
**Cardiotoxicity**	0.0165
**Nephrotoxicity**	0.0165
**Embryonic Stem Cell Differentiation**	0.0169
**Adipogenesis**	0.0258
**Chemosensitivity of Tumor Cells**	0.0263
**Autophagy**	0.0263
**Toxicity**	0.028
**Cholesterol Homeostasis**	0.0331

Supplementary Table S5. miRNA enrichment for the AraC drug release in SEM. Based on a p-value of $p < 0.05$ the miRNA enrichment was used to identify disease and functional annotation for the AraC drug release in SEM.

**Disease/ Ontology**	**adjusted p-value**
**Lymphoma, Large B-Cell, Diffuse**	1.00E-02
**Leukemia, Myeloid, Acute**	0.0113
**Leukemia**	0.0457
**Skeletal Muscle Cell Differentiation**	9.81E-05
**Apoptosis**	1.02E-03
**Autophagy**	3.84E-03
**Tumor Suppressor miRNAs**	6.60E-03
**Response to Hypoxia**	8.80E-03
**Epithelial-to-Mesenchymal Transition**	0.0157
**Nephrotoxicity**	0.0175
**Smooth Muscle Cell Proliferation**	0.0195
**Multiciliated Cell Differentiation**	0.0246
**Muscle Development**	0.0263
**Peritoneal Cavity Homeostasis**	0.0312
**Osteogenesis**	0.0329
**Actin Filament Network Formation**	0.0367

**Aging**	0.039
**Inflammation**	0.0425

Supplementary Table S6. miRNA enrichment for the Dexa drug release in SEM. Based on a p-value of $p < 0.05$ the miRNA enrichment was used to identify disease and functional annotation for the Dexa drug release in SEM.

**Disease/ Ontology**	**adjusted p-value**
**Lymphoma, Primary Effusion**	1.39E-09
**Thyroid Lymphoma**	8.56E-05
**Leukemia, Myeloid, Chronic**	3.29E-04
**Lymphoma**	1.22E-03
**Lymphoma, Extranodal NK-T-Cell**	1.31E-03
**Leukemia, Myeloid, Acute**	2.36E-03
**Lymphoma, Mantle-Cell**	6.31E-03
**Lymphoma, Hodgkin**	0.0103
**Lymphoma, Large-Cell, Anaplastic**	0.0256
**Leukemia, Lymphoblastic, Acute, B-Cell**	0.0299
**Lymphoma, Large B-Cell, Diffuse**	0.045
**Innate Immunity**	2.93E-09
**Tumor Suppressor miRNAs**	1.87E-08
**Folliculogenesis**	1.29E-06
**Aging**	2.44E-06
**Cell Differentiation**	1.03E-05
**Adipocyte Differentiation**	1.23E-05
**Cell Division**	1.59E-05
**Ovarian Follicle Development**	3.47E-05
**Hepatic Stellate Cell Differentiation**	8.56E-05
**Neural Stem Cell Differentiation**	1.69E-04
**T-Cell Differentiation**	2.56E-04
**Chromatin Remodeling**	2.91E-04
**Adipogenesis**	6.42E-04
**Cell Death**	8.54E-04
**Cardiac Regeneration**	9.41E-04
**Epithelial-to-Mesenchymal Transition**	1.25E-03
**Glucose Metabolism**	2.41E-03
**Cell Proliferation**	5.45E-03
**Hematopoiesis**	5.95E-03
**Cell Cycle**	6.55E-03
**Skeletal Muscle Cell Differentiation**	0.0103
**Immune Response**	0.0108
**Anti-Cell Proliferation**	0.0115

**Peritoneal Cavity Homeostasis**	0.0117
**Inflammation**	0.027
**Retinal Pigment Epithelium Development**	0.0425
**Angiogenesis**	0.0463
**Latent Virus Replication**	0.0495
